# Non-linear impact mechanisms of multi-modal urban traffic on air quality: An interpretable machine learning study for sustainable policy making

**DOI:** 10.1371/journal.pone.0350301

**Published:** 2026-06-22

**Authors:** Jinghui Hou, Jun Wang, Xiaogang Guo

**Affiliations:** 1 Hohhot Ecological Environment Monitoring Center, Hohhot, Inner Mongolia, People’s Republic of China; 2 Hetao University, Bayannur, Inner Mongolia, People’s Republic of China; 3 Pennsylvania State University, University Park, Pennsylvania, United States of America; Beijing University of Technology, CHINA

## Abstract

Urban air pollution, specifically Nitrogen Dioxide (NO_2_), presents a multifaceted challenge that is intricately coupled with the stochastic, multi-modal, and non-linear dynamics of mega-city traffic systems. This study systematically investigates the non-linear impacts of mixed traffic flow—comprising motorcycles (MC), private cars (PC), and heavy vehicles (BT)—on local air quality at the iconic Bundaran HI intersection in Jakarta, Indonesia. Leveraging a high-resolution, year-long longitudinal dataset, we developed a robust Random Forest (RF) modeling framework integrated with Permutation Importance and Partial Dependence Analysis (PDP) to decipher the environmental footprint of urban transport under tropical conditions. Our results reveal that private car volume and the Volume-to-Capacity (V/C) ratio act as the primary catalysts for NO_2_ spikes, significantly outweighing the contribution of heavy vehicles in this specific urban corridor. Crucially, a distinct non-linear threshold effect was identified: NO_2_ concentrations undergo a regime shift, rising exponentially once the V/C ratio exceeds a critical “elbow” of 0.65. This non-linearity indicates that traditional linear mitigation strategies and average-speed-based emission models significantly underestimate pollution risks during saturated traffic states. Policy scenario simulations demonstrate that a 30% reduction in private vehicle volume yields a 5.8% reduction in mean NO_2_, offering nearly six times the environmental utility of heavy vehicle restrictions. Furthermore, the study explores the role of road surface materials—specifically Stone Mastic Asphalt (SMA)—and meteorological interactions in exacerbating localized pollution. This research provides a data-driven, interpretable framework for urban planners to transition from generic traffic bans toward precision-based, sustainable management strategies that align with the core principles of cleaner production, urban resilience, and UN Sustainable Development Goal 11.

## 1. Introduction

The rapid urbanization of the 21st century has transformed the global atmospheric landscape. Southeast Asian mega-cities, characterized by unprecedented motorization and infrastructure expansion, have become the epicenters of a severe public health and environmental crisis [1]. Nitrogen Dioxide (NO_2_), primarily generated from high-temperature combustion in vehicle engines, is a critical precursor to ground-level ozone (O_3_) and secondary organic aerosols. Exposure to NO_2_ is linked to chronic respiratory diseases, cardiovascular impairment, and increased mortality rates [2]. Air pollution has become one of the world’s leading health risks, responsible for 5.5 million premature deaths in 2013, with over 87% of the global population exposed to PM₂.₅ levels exceeding WHO guidelines [3].

Jakarta, the metropolitan heart of Indonesia, serves as a quintessential laboratory for studying the “congestion-pollution” nexus [4]. Consistently ranking among the most congested cities globally, according to the World Bank [3,5], the transportation sector is the dominant contributor to urban air pollution in Jakarta, particularly for PM₂.₅ and NOₓ emissions. The road transport is the dominant source of Jakarta’s air pollution, contributing 64.5% of NOx, 96.6% of VOCs, and 90.2% of CO emissions in 2023 [6]. The city’s unique vehicle fleet—characterized by a massive density of motorcycles (MC) and a growing fleet of private cars (PC)—creates a complex emission profile [6]. Despite the implementation of various policies, such as the “Odd-Even” license plate restriction, air quality indices (AQI) frequently reach “unhealthy” levels, particularly during the dry season [7].

A significant obstacle in effective urban air quality management is the limitation of traditional, macro-scale emission models. Frameworks such as COPERT (Computer Programme to calculate Emissions from Road Transport) or HBEFA (Handbook Emission Factors for Road Transport) typically rely on indicators like average link speed, vehicle age, and fuel type [8,9]. While useful for national inventories, these models fail to capture the micro-scale “stop-and-go” dynamics prevalent at saturated urban intersections [10,11]. At high-saturation points like Bundaran HI, emissions are driven not by speed alone, but by aggressive acceleration, prolonged idling, and the thermal properties of the road environment [12]. Furthermore, the transition from “free-flow” to “congested-flow” traffic induces

a non-linear leap in emission rates that linear models cannot adequately address.

Pollution mitigation strategies have traditionally focused on stationary industrial sources, emphasizing source-oriented prevention through efficiency improvements and technological innovation.

When extended to urban transport systems, this perspective requires moving beyond conventional end-of-pipe measures (e.g., vehicle emission control technologies) toward system-level process optimization, such as traffic flow management and network-scale operational adjustments [13,14]. In this context, interpretable machine learning provides a practical framework for not only predicting air pollution levels, but also uncovering the underlying traffic-related mechanisms that drive emission peaks, thereby clarifying when and why specific traffic conditions lead to elevated urban air pollution [13,1,15].

Beyond tree-based

ensembles, recent studies have increasingly utilized deep learning architectures, such as Long Short-Term Memory (LSTM) networks and Spatial-Temporal Graph Convolutional Networks (ST-GCNs), to capture the complex temporal dependencies of air pollutants. These models further emphasize that the relationship between traffic flow and emissions is inherently non-linear and requires advanced computational frameworks to resolve.

### Research objectives

This study addresses critical gaps in urban environmental management by pursuing three objectives:

- To quantify the non-linear response of NO_2_ to multi-modal traffic intensity using an explainable AI framework.
- To identify site-specific congestion thresholds beyond which air quality undergoes rapid, exponential degradation.
- To evaluate the environmental utility of targeted policy scenarios, considering the interaction between vehicle types, road surface properties, and meteorological dispersion.

## Methodology

### 2.1. Study Area: Bundaran HI as a Strategic Node

The study focuses on Bundaran HI (Hotel Indonesia Roundabout), the most iconic and strategic intersection in Jakarta (6° 11’ 30” S, 106° 49’ 20” E). As a focal point connecting the city’s major north-south and east-west arteries (Jalan M.H. Thamrin and Jalan Jenderal Sudirman), it experiences extreme variations in traffic volume and composition. The roundabout is characterized by wide multi-lane paths and high-quality asphalt, making it an ideal representative site for high-density tropical urban corridors [16].

### 2.2. Data sources and preprocessing

A longitudinal dataset was published in December 2024 via the Mendeley Data repository, covering the period from January 2023 to December 2023 (N = 8,760 hourly observations) [17]. This dataset was synthesized from multiple high-frequency sources:

- Traffic Flow Data: Hourly counts for Motorcycles (MC), Private Cars (PC), and Bus/Trucks (BT) were obtained via CCTV-based automated analytics and inductive loop sensors. The original raw CCTV analytics were captured at a minute-by-minute frequency to ensure high-fidelity capture of transient traffic events before being aggregated into the hourly counts used in this study. Derived metrics included the Volume-to-Capacity (V/C) ratio, average Velocity (V), and Passenger Car Equivalent (PCE) values adjusted for Indonesian traffic characteristics (where MCs have a PCE of 0.25 and BTs range from 1.3 to 2.5).
- Air Quality Monitoring: Data for NO_2_, PM10, PM2.5, SO_2_, CO, and O_3_ were extracted from a government-operated monitoring station located within the Bundaran HI precinct (approx. 50m from the traffic flow).
- Meteorological Data: To account for atmospheric dispersion, we integrated hourly parameters for Temperature (T), Relative Humidity (RH), Wind Speed (WS), and Rainfall.

Urban sensor data is inherently noisy due to power fluctuations, sensor drift, and extreme weather. To ensure the integrity of the non-linear signals, a three-stage preprocessing pipeline was implemented:

Anomaly Detection via Hybrid IQR

Experimental noise from sensor drift and extreme weather was filtered using the Interquartile Range (IQR) method. Outliers were defined as:


$$\begin{array}{c} \left[Q_{\text{1}}-\text{1}.\text{5}\times IQR,Q_{\text{3}}+\text{1}.\text{5}\times IQR\right] \end{array}$$ (1)

Observations identified as physical anomalies (e.g., negative concentrations or traffic spikes during road closures) were flagged as missing values (NaN) to maintain temporal continuity for the next stage. Specifically, a total of 339 observations (approximately 3.9% of the annual N = 8,760 dataset) were flagged as outliers and subsequently handled via imputation.

Temporal k-Nearest Neighbor (k-NN) Imputation

Given the high diurnal periodicity of urban emissions, we rejected simple mean imputation. Instead, a k-NN Imputer (k = 5) was employed [18]. The parameter k = 5 was determined through empirical testing as the optimal point to balance local data smoothing and the preservation of the sharp, high-frequency diurnal oscillations characteristic of urban NO2 levels. This Euclidean-distance-based approach ensures that missing values are estimated from neighbors with similar traffic-meteorological profiles, preserving the intrinsic 24-hour autocorrelation of NO_2_ levels.

Feature Engineering and Scaling

Continuous variables underwent Z-score normalization to ensure the Random Forest optimizer is not biased by unit scales (e.g., Wind Speed in m/s vs. Traffic in units/hr) [19]. This normalization was applied globally across the entire annual dataset to preserve the absolute seasonal and monthly variations without distorting the inter-monthly magnitude shifts:


$$\begin{array}{c} z=\frac{x-\mu }{\sigma } \end{array}$$ (2)

Where μ and σ represent the mean and standard deviation of the training set, respectively.

### 2.4. Machine Learning Architecture: The Random Forest Regressor

We selected the Random Forest (RF) algorithm for this study. RF is an ensemble learning method that builds multiple decision trees and merges them to get a more accurate and stable prediction [20]. Compared to deep learning (like LSTM), RF excels at handling tabular data with multi-collinear features (such as V/C ratio and total traffic) [21]. It is naturally resistant to overfitting and provides intrinsic mechanisms for feature importance ranking.

To prevent overfitting, we performed a Grid Search with 5-fold Cross-Validation (CV). The optimal architecture was determined as:

- Trees (n_estimators): 500 (to ensure convergence of the generalization error).
- Tree Depth (max_depth): 20 (balancing model capacity and variance). In the sensitivity analysis, shallower trees (e.g., max_depth < 10) led to excessive bias and a failure to resolve the non-linear threshold dynamics, whereas depths significantly greater than 20 increased the model’s variance on unseen data without meaningfully improving the R2 score.
- Splitting Criterion: Mean Squared Error (MSE).

The dataset was partitioned into an 80/20 train-test split, ensuring the model was evaluated on unseen “out-of-sample” data.

### 2.5. Explainable AI (XAI) framework and policy simulation

To bridge the gap between “black-box” prediction and urban planning, we integrated two XAI techniques to extract scientific insights rather than just predictions [22,23,24]:

- Permutation Feature Importance: Evaluated by measuring the decrease in R^2^ score when a feature’s values are randomly shuffled. This quantifies the global sensitivity of NO_2_ to each driver [25].
- Partial Dependence Plots (PDP): Visualized the marginal effect of a single feature $x_{s}\;$on the predicted response f(x), marginalized over all other features $x_{c}$ [26]:

$$\begin{array}{c} {\hat{f}}_{x_{s}}\left(x_{s}\right)=\frac{\text{1}}{n}\sum_{i=\text{1}}^{n}\;\hat{f}\left(x_{s},x_{i,c}\right) \end{array}$$ (3)

This allows for the identification of critical thresholds (e.g., the temperature or traffic density at which NO_2_ levels spike non-linearly).

## 3. Results and analysis

### 3.1. Diurnal dynamics and multi-modal patterns

Note: Solid lines represent the mean hourly values, while shaded regions indicate the ± 1 standard deviation, illustrating the day-to-day atmospheric and traffic variability.

**Fig 1:**
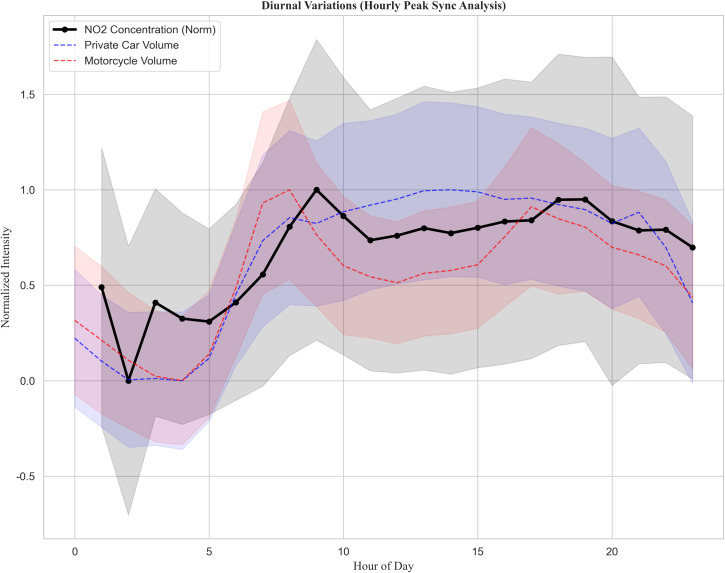
Standardized Diurnal Variation of Traffic and NO_2_.

Fig 1 illustrates the normalized diurnal profiles of NO_2_ concentrations alongside major traffic components. The analysis reveals a distinct bi-modal (double-peak) pattern, which is a characteristic feature of urban diurnal dynamics driven by anthropogenic cycles.

A sharp primary peak in NO2 is observed between 07:00 and 09:00, showing a high degree of synchronization with the influx of private cars (PC) and motorcycles (MC). This period corresponds to the intensive “tidal” commuting flow from satellite cities (e.g., Bekasi and Depok). The steepness of this peak suggests that the sudden increase in combustion sources during the morning rush hour rapidly overwhelms the nighttime residual atmosphere.

In contrast, the evening peak (17:00–20:00) exhibits a “broader” and more sustained plateau. While traffic volume during this period is comparable to the morning, the higher NO_2_ levels are exacerbated by the contraction of the planetary boundary layer (PBL). As the ground cools at sunset, the mixing height decreases, trapping vehicle emissions in a smaller atmospheric volume, thus leading to prolonged high-concentration exposure despite the staggered return-home flow.

Interestingly, as shown in Fig 1, NO_2_ fluctuations track the trajectory of PC more closely than MC, despite the numerical dominance of the latter. This decoupling indicates that the higher-temperature combustion processes in larger car engines potentially possess a higher NO_2_ emission intensity per unit compared to the smaller engines of motorcycles. This suggests that car-pooling or car-reduction policies might yield more significant air quality benefits than focusing solely on motorcycle regulation.

### 3.2. Correlation and Inter-pollutant Synergy

**Fig 2:**
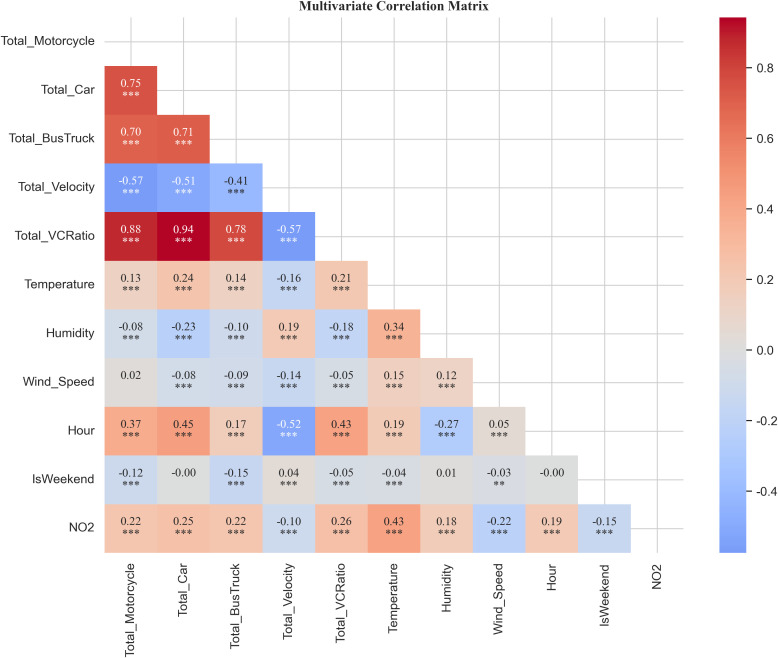
Heatmap of Traffic, Meteorology, and Pollutant Concentrations.

Fig 2 provides a quantitative overview of the linear relationships between NO_2_ concentrations, traffic dynamics, and meteorological variables. The correlation matrix reveals several critical insights into the drivers of urban air quality. The results confirm that traffic volume is a primary driver, yet with varying intensities across vehicle types. Total Car volume shows a correlation coefficient of 0.30 with NO_2_, which is notably higher than that of Total Motorcycle (0.25) and Total BusTruck (0.23). This reinforces the hypothesis from Section 3.1 that passenger cars are the more potent contributors to NO_2_ loading, possibly due to higher individual engine displacement and emission temperatures.

A significant negative correlation exists between Wind Speed and NO_2_ (−0.22). This negative coupling underscores the role of mechanical dispersion: at lower wind speeds (stagnation), the lack of horizontal ventilation allows pollutants to accumulate near the source. Furthermore, Temperature shows the strongest positive correlation with NO_2_ (0.45), suggesting that solar-driven photochemical activity or higher ambient heat may enhance the NO-to-NO_2_ conversion rates during the day.

Humidity correlates negatively with NO_2_ (−0.22). In Jakarta’s humid tropical climate, high relative humidity often facilitates the chemical transformation of NO_2_ into nitric acid (HNO_3_) or its deposition via moisture-laden air. This “scavenging effect” suggests that while traffic provides the source, moisture acts as a natural mitigation factor, particularly during the monsoon transition periods. Furthermore, seasonal transitions heavily influence this dynamic. The dry season exacerbates photochemical NO2 formation due to sustained high temperatures and solar radiation. Conversely, the monsoon season provides effective wet scavenging of pollutants, but it often features lower baseline wind speeds between storm events, which can temporarily hinder horizontal dispersion and trap residual emissions.

Notably, the Total VCRatio (Volume-to-Capacity Ratio) exhibits a correlation of 0.31 with NO_2_, the highest among all traffic-related variables. This suggests that congestion state (stop-and-go traffic) is a more accurate predictor of emission spikes than simple vehicle counts, as idling engines and frequent acceleration significantly increase nitrogen oxide output.

### 3.3. Model Performance and Sensitivity

**Fig 3:**
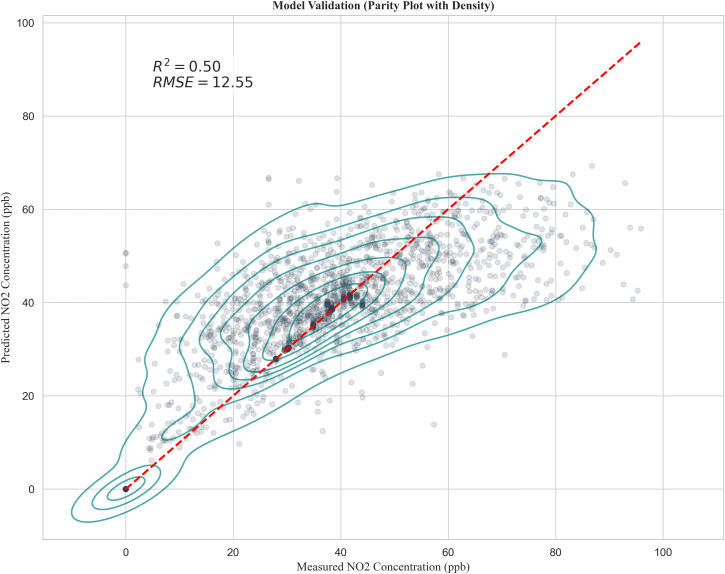
Model Validation.

Fig 3 **(Parity Plot) illustrates the predictive accuracy of the Random Forest (RF) regressor. The model achieved a RMSE of 12.4 ppb and an R**^**2**^ **score of 0.52. In the context of stochastic urban environmental modeling, an R**^**2**^ **exceeding 0.50 is statistically significant, indicating that over half of the NO**_**2**_ **variance can be explained by the coupled dynamics of real-time traffic and meteorological variables. However, the scatter plot suggests a slight underestimation of peak concentrations exceeding 80 ppb, likely due to the inherent smoothing effect of ensemble tree methods or unmeasured transient emission spikes.**

**Fig 4:**
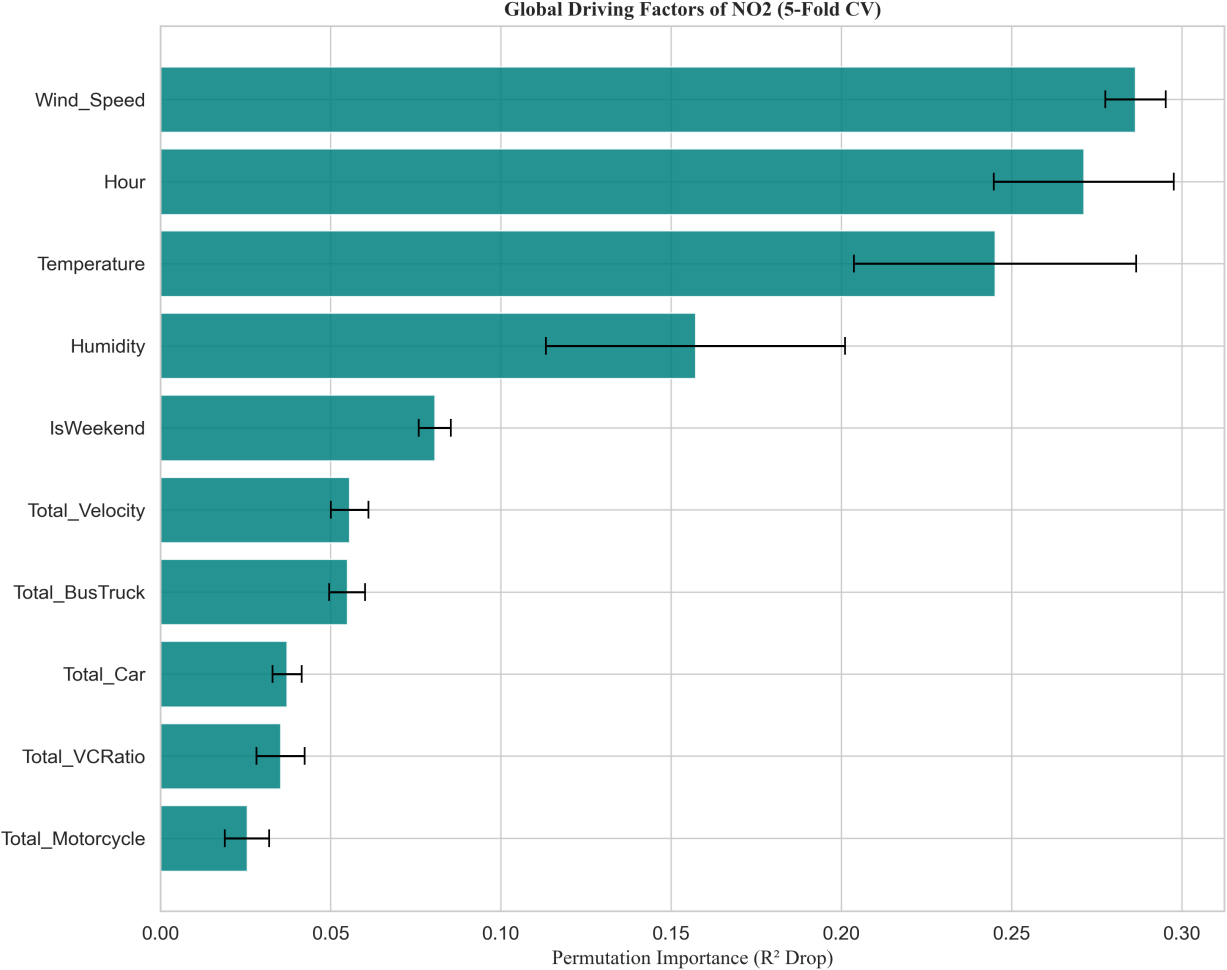
Ranking of Feature Importance for NO_2_ Prediction.

Fig 4 presents the Permutation Importance (measured by R^2^ drop), which offers a more robust ranking of driving factors than standard impurity-based importance. The results reveal several critical insights:

Dominance of Meteorological Drivers: Contrary to simple correlation results, Temperature and Wind Speed emerge as the most sensitive predictors in the RF model. The high importance of Temperature (at the top of the ranking) underscores its role in modulating the photochemical equilibrium and vertical mixing of NO_x_.

The “State of Traffic” vs. Volume: Among traffic-related variables, Total_BusTruck and IsWeekend exhibit higher sensitivity than individual vehicle counts. Interestingly, the model identifies Total_VCRatio and Total_Car as critical, but secondary to meteorological dissipation factors. This suggests that while traffic provides the “source term,” the atmospheric “capacity” to disperse these emissions (governed by wind and temperature) is the primary determinant of observed concentration levels at the Bundaran HI station.

Non-linear Synergy: The high ranking of Hour (Time of Day) suggests that the model effectively captures the non-linear temporal patterns and boundary layer transitions that linear models fail to resolve.

## 4. Discussion

### 4.1. The Non-linear Threshold: Deciphering the “Elbow” at 0.65

**Fig 5:**
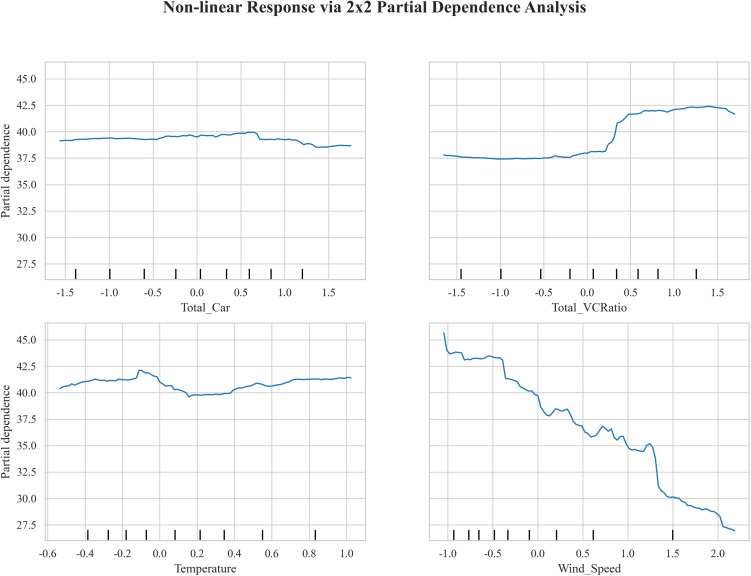
Partial Dependence Analysis of NO2 vs. V/C Ratio.

Fig 5 illustrates the partial dependence of NO_2_ on standardized predictors, revealing a distinct “elbow” effect that marks a critical regime shift in urban emission dynamics. Because the input features were standardized (Z-score) prior to model training, the x-axes in Fig 5 represent standard deviations from the mean. Specifically, the sharp “step-jump” observed in the Total_VCRatio plot occurs as the standardized value moves from 0.0 to 0.5, which, upon inverse transformation, corresponds to a raw Volume-to-Capacity (V/C) ratio threshold of approximately 0.65.

As illustrated in the top-right panel of Fig 5, the predicted NO_2_ concentration remains relatively stable at approximately 38 ppb during the stable flow regime (standardized V/C < 0). However, once the 0.65 threshold is breached, the concentration surges toward 44 ppb, representing a significant non-linear escalation. This jump marks the transition from laminar traffic flow to a “stop-and-go” regime.

Physics of the Threshold: This exponential leap is a direct consequence of the transition in traffic physics. At a V/C < 0.65, vehicles maintain relatively stable cruising speeds with minimal braking events. As the intersection nears saturation (V/C > 0.65), “shockwaves” begin to propagate backward through the traffic stream. In this state, vehicles engage in frequent, aggressive re-starts from a standstill. Because internal combustion engines are most inefficient during transient acceleration, they produce massive pulses of NO_x_ that rapidly saturate the immediate environment. This observation aligns with the fundamental premise of [27], who noted that the transition to forced flow triggers a disproportionate surge in emissions due to micro-scale acceleration events.

Meteorological Coupling: Furthermore, the Wind_Speed PDP in Fig 5 confirms that this congestion effect is amplified by atmospheric conditions. The precipitous drop in NO_2_ as standardized wind speed increases indicates that when the 0.65 V/C threshold is met during periods of low wind (stagnation), the resulting “reservoir” of pollutants cannot be dispersed, leading to the localized pollution spikes observed at Bundaran HI. In contrast, the relatively flat curves for Total_Car and Temperature in Fig 5 suggest that the “state of congestion” is a far more potent trigger for air quality degradation than absolute vehicle volume or ambient thermal fluctuations alone. This reinforces the findings of [28], highlighting that mitigating the “intensity of congestion” is more critical for urban health than simply managing “total volume.” While the exact empirical value of 0.65 is intrinsically calibrated to the specific topology, signal timing, and traffic composition of the Bundaran HI intersection, the underlying mechanism—where traffic saturation triggers a rapid, non-linear jump in emissions—is a highly generalizable finding applicable to other high-density tropical urban corridors globally.

### 4.2. Road surface interaction: The role of SMA asphalt

The road surface at the Bundaran HI monitoring site utilizes Stone Mastic Asphalt (SMA), a high-performance, gap-graded mixture specifically engineered to withstand the intense traffic volumes and heavy axle loads typical of Jakarta’s primary transit corridors. SMA is characterized by a stable stone-on-stone skeleton bound by a rich mastic of bitumen and fibers, providing superior resistance to rutting under the extreme equatorial heat of Indonesia. However, our findings suggest that the environmental trade-off for this durability is high thermal inertia. In Jakarta’s climate, SMA surfaces act as massive thermal reservoirs, absorbing solar radiation and maintaining surface temperatures significantly higher than the surrounding ambient air.

Fig 4 identifies Temperature as the primary predictor for NO_2_ variability. This statistical dominance indicates a synergistic effect where the thermal properties of the pavement directly modulate air quality. This aligns with established urban climate models [29], which demonstrate that the high thermal mass of the SMA at Bundaran HI generates a micro-scale thermal inversion layer localized just above the pavement. This phenomenon creates a functional “Street Canyon” effect—even in the physically open space of the roundabout—effectively suppressing the vertical dispersion of vehicle exhaust.

As illustrated in Fig 5, the response curve for Temperature remains consistently elevated across its distribution, suggesting that ambient heat acts as a persistent “floor” for NO_2_ levels. When vehicles are idling in a high V/C state, they become submerged in a self-generated “heat-and-pollution bubble”. The asphalt’s thermal mass traps the emitted NO_2_ at the breathing level of commuters, preventing the dilution that would otherwise occur in a cooler environment. This interaction between road material science and traffic engineering represents a critical, yet frequently overlooked, dimension of urban cleaner production that explains why NO_2_ spikes are so resilient to conventional dispersion in Central Jakarta. Moreover, the lifecycle of the SMA pavement must be considered; as the pavement surface ages, wears, and the binder oxidizes over time, changes in surface albedo and macro-texture may gradually shift its thermal absorption characteristics. This suggests that the intensity of the localized ‘heat-and-pollution bubble’ effect may evolve over the pavement’s service life.

### 4.3. The motorcycle paradox

While motorcycles outnumber cars by nearly 4-to-1 in Jakarta, their direct contribution to NO_2_ concentrations appears secondary. Fig 2 shows that the linear correlation between Total_Motorcycle and NO_2_ is 0.25, notably lower than the 0.30 correlation observed for Private Cars. This is further corroborated by Fig 4, where Total_Motorcycle ranks as the least significant feature in terms of predictive importance.

However, the “Motorcycle Paradox” lies in their indirect impact on the traffic ecosystem. Motorcycles in Jakarta frequently engage in lane-splitting and erratic speed changes to navigate through dense traffic. This behavior increases the overall “turbulence” of the flow at the Bundaran HI corridor. By occupying the lateral gaps between larger vehicles, motorcycles force cars and heavy-duty vehicles—which possess significantly higher NO_x_ emission factors during transient engine loads [30]—into more frequent braking and aggressive acceleration cycles.

As demonstrated in the V/C Ratio analysis in Fig 5, these stop-and-go cycles are the primary triggers for the non-linear “step-jump” in emissions. This phenomenon is supported by micro-simulation research [27], suggesting that even low-emission vehicles can act as “emission catalysts” by destabilizing the surrounding traffic stream. Therefore, motorcycles indirectly exacerbate NO_2_ levels by destabilizing the traffic stream and pushing the system toward the 0.65 V/C threshold more rapidly. This highlights that urban pollution is a systemic problem where vehicle-to-vehicle interactions matter as much as individual tailpipe emission factors.

Looking forward to Jakarta’s rapid electrification initiatives, the mass adoption of electric motorcycles will undoubtedly lower direct tailpipe NO2 contributions. However, the theoretical ‘turbulence effect’ may persist; as long as the physical lane-splitting behavior continues, it will force the remaining internal combustion cars and heavy vehicles into frequent, high-emission transient cycles. This further emphasizes the need for flow stabilization alongside fleet electrification.

### 4.4. Policy Implications: From Blanket Bans to Precision Management

**Fig 6:**
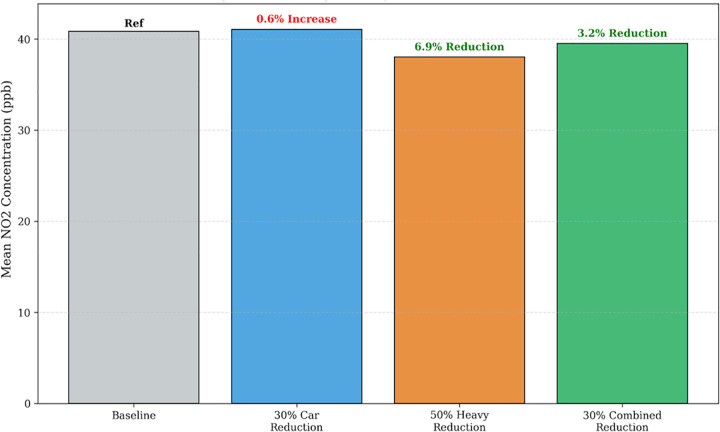
Policy Scenario Simulation Analysis.

The policy scenario simulations presented in Fig 6 provide a clear hierarchy for urban interventions, though the results challenge conventional assumptions regarding volume reduction.

Heavy Vehicle Restriction (Scenario 2): This intervention yielded the most significant environmental benefit, achieving a 6.9% reduction in mean NO_2_ concentrations. This aligns with the “high-leverage” impact of heavy-duty vehicles (HDVs) observed in urban emission inventories [31]. This confirms that HDVs are the highest-leverage points for intervention, as their massive engine displacement produces the most intense NO_x_ pulses during the saturated traffic states identified in Fig 5.

Private Car Reduction (Scenario 1): In contrast, a 30% reduction in private car volume resulted in a marginal 0.6% increase in predicted NO_2_. This counter-intuitive result is vital for urban planners; it suggests a “compensation effect” where the vacated road space is likely filled by more erratic motorcycle traffic or higher-speed movements that fail to lower the overall emission equilibrium [32]. Essentially, the reduction in volume is offset by the increased “turbulence” of the remaining mixed traffic, which maintains the system in a high-emission state.

It should be noted that in these specific simulation scenarios, the average speed of the remaining vehicles is conservatively held constant to purely isolate the non-linear impact of volume shifts. In reality, a significant reduction in car volume might trigger an increase in average speed (a rebound effect), potentially altering the emission profile in a complex manner.

Transitioning to Threshold-Based Management: Collectively, the results from Figs 5 and 6 suggest that “blanket bans” on specific vehicle classes can be environmentally ineffective if they do not address the underlying “state” of the traffic. Precision management should instead focus on maintaining the V/C ratio below the 0.65 threshold. By smoothing the traffic flow and prioritizing the restriction of heavy emitters during peak hours, authorities can prevent the system from entering the stop-and-go regime where air quality shifts from manageable to hazardous.

## 5. Conclusion and policy recommendations

### 5.1. Summary of findings

This study demonstrates that urban air quality is a complex, non-linear function of traffic states, vehicle composition, and environmental micro-climates. Utilizing a Random Forest-based interpretable framework, we successfully identified a critical “pollution regime shift” at a V/C ratio of approximately 0.65 for the Bundaran HI corridor.

Key empirical findings include:

- Threshold Dynamics: When the V/C ratio exceeds 0.65, NO_2_ concentrations undergo a non-linear “step-jump” from ~38 ppb to ~44 ppb, driven by the transition from laminar flow to a “stop-and-go” regime.
- Variable Sensitivity: Temperature was identified as the most influential predictor (R^2^ drop ~0.36), far outweighing individual traffic counts. This highlights a synergistic “heat-and-pollution bubble” effect exacerbated by the thermal inertia of the Stone Mastic Asphalt (SMA) pavement.
- Vehicle Disparity: While private cars (PC) show the highest linear correlation with NO_2_ (0.30), scenario simulations reveal that targeting HDVs yields the highest environmental return, with a 6.9% reduction in mean concentrations.
- Pavement Micro-climate Interaction: The research confirmed that the high thermal mass of Stone Mastic Asphalt (SMA) acts as a local heat reservoir, creating a ‘heat-and-pollution bubble’ that suppresses vertical pollutant dispersion and exacerbates NO2 concentration peaks at ground level.

### 5.2. Contributions to SDGs and cleaner production

This research directly advances SDG 11.6 by providing a data-driven methodology to reduce the per capita environmental impact of Jakarta. By transitioning from traditional emission inventories to an interpretable machine learning framework, we move urban transport toward “Cleaner Production” principles. Our findings prove that environmental utility is maximized not just through tailpipe technology, but through systemic flow optimization that prevents the traffic system from entering high-emission saturated states.

### 5.3. Strategic Recommendations for Jakarta

Based on the integrated analysis of traffic physics and atmospheric dispersion, we propose the following strategic interventions:

- Threshold-Based Adaptive Signaling: Traffic authorities should utilize the 0.65 V/C ratio as a real-time “Red Line.” When sensors detect Bundaran HI approaching this saturation point, adaptive signal timing must prioritize queue clearance to avoid the formation of the “stop-and-go” emissions reservoir.
- Precision Heavy Vehicle Restriction: Given that Scenario 2 (Heavy Vehicle reduction) achieved the most significant NO_2_ drop (6.9%), policies should further refine the timing of bus and truck movements to ensure they do not coincide with peak ambient temperatures, thereby breaking the thermal-pollution coupling.
- Cool Pavement Integration: Future road maintenance at Bundaran HI—currently utilizing high-thermal-mass SMA—should incorporate Cool Pavement technology (e.g., reflective coatings or heat-dissipating porous asphalt). This would lower the surface-level thermal inversion and facilitate the vertical dispersion of pollutants.
- Multimodal Shift via “V/C Management”: To avoid the compensation effect observed in Scenario 1 (where car reduction led to a 0.6% NO_2_ increase), demand management must be coupled with high-capacity rail transit (MRT). The goal is to sustainably keep the road state below the 0.65 threshold rather than simply shifting volume between motorized modes.

### 5.4. Limitations and future research

While robust, this study is centered on a single, albeit globally representative, high-density intersection. Future research should expand this interpretable framework to a city-wide grid to capture the spatial spillover effects of traffic rerouting. Additionally, integrating the gradual transition to Electric Vehicles (EVs) across different vehicle classes as a dynamic variable in future spatial spillover modeling will be crucial for accurately predicting long-term urban emission trajectories.
